# Disease Mongering: One of the Hidden Consequences

**DOI:** 10.1371/journal.pmed.0030316

**Published:** 2006-07-25

**Authors:** Kenneth Gillman

**Affiliations:** **1**Pioneer Valley Private HospitalMackayAustralia

I found Moynihan and Henry's article on disease mongering [ 1] interesting, especially because I have previously suggested that the medical profession might consider being more proactive concerning various problematic areas in their interactions with the pharmaceutical industry by exercising their considerable power and improving the scientific quality of research [ 2]. There is a strong tendency for doctors to be trusting and accepting of the good intentions and honesty of others. It takes a substantial amount of evidence for doctors to adopt the contrary posture of distrust. Perhaps the profession is, understandably, at that point with pharmaceutical companies.

A significant hidden area related to disease mongering is the inevitable increase in doctors' medico-legal insurance costs. The pharmaceutical industry has generally been quite successful in getting doctors to shoulder the blame for the negative consequences of drug treatment. They are quick to inform the profession (and patients, by covert direct-to-consumer advertising) of any evidence favourable to the promotion of their drug, but slow to update the product information, or inform doctors, about side effects, complications, or drug interactions [ 3]. It is dishonest to actively promote supposed advantages (to patients) whilst consciously failing to look for, or alert doctors to, the disadvantages. Furthermore, inducing patients to visit doctors and pressure them into colluding with drug company advertising is a subtle form of bullying.

Medical insurers tend to accept full responsibility on behalf of doctors without much attempt to bring others into legal actions, especially drug companies. Since both patients and doctors are being fed misinformation, it may be that a greater part of the responsibility for difficulties should be apportioned to drug companies. Perhaps both doctors and drug companies need to be reminded that only doctors are able to sign prescriptions and take the primary responsibility for the consequences. It may be time for medical organizations and authorities to impose conditions and demand more information from pharmaceutical companies if they are going to agree to sign the script. As but one of many possible examples, how many doctors realise that drug toxicity data are rarely made available and that much of the data presented to regulatory authorities are not available to ordinary doctors? I suggest that those in the profession who are in a position to influence such matters should give serious consideration to these and other similar questions and exercise their power.
